# Evaluating the health promotion effects of the IKAP care model in older adults with periodontitis

**DOI:** 10.1097/MD.0000000000044034

**Published:** 2025-09-05

**Authors:** Yeqian Zhu, Jing Qian, Wen Ma, Guojun Miao, Yubo Xu

**Affiliations:** a Department of Stomatology, Shanghai East Hospital, School of Medicine, Tongji University, Shanghai, Shanghai, China.

**Keywords:** geriatric dentistry, health promotion, oral health, periodontitis, quality of life

## Abstract

This study assessed the impact of the information–knowledge–attitude–practice (IKAP) care model on health promotion among elderly patients diagnosed with periodontitis. Using a retrospective case-control design, 131 patients aged 60 years or older, treated between June 2022 and June 2024, were categorized into 2 groups: the IKAP care group (n = 68) and the conventional care group (n = 63). Clinical outcomes, behavioral modifications, and quality of life were evaluated through periodontal examinations, health knowledge questionnaires, and standardized assessments. Initial results at 1 and 3 months post-intervention revealed no significant differences in periodontal health between the 2 groups. However, by the 6-month follow-up, the IKAP group demonstrated superior improvements in gingival bleeding index, probing depth, and clinical attachment level compared to the conventional group. Additionally, a higher proportion of patients in the IKAP group achieved sustained behavioral changes over time. Both short- and long-term assessments indicated that the IKAP model significantly enhanced oral health self-efficacy and overall quality of life, as reflected in lower oral health impact profile (OHIP-14) scores. These findings suggest that the IKAP care model effectively promotes oral health, fosters positive health behaviors, and improves self-management capabilities in elderly periodontitis patients. The study highlights the potential of the IKAP approach as a comprehensive strategy for geriatric oral health management, offering valuable insights for clinical practice.

## 1. Introduction

With the global population aging, the health challenges faced by elderly individuals have garnered increasing attention. According to the World Health Organization, people aged 65 and older now make up 13.1% of the global population, with this proportion projected to rise to 16.5% by 2050.^[1]^ This demographic shift poses significant challenges to public health systems worldwide and has stimulated research into better health management strategies for the elderly.^[2]^ Among the various age-related chronic conditions affecting older adults, periodontitis is a common oral disease that significantly impacts the quality of life and overall health of the elderly.^[3]^

Periodontitis is a chronic inflammatory disease primarily caused by bacterial infection, characterized by symptoms such as swollen, bleeding gums, tooth mobility (TM), and eventual tooth loss. It is closely associated with factors like poor oral hygiene, declining immune function, and comorbidities such as diabetes and cardiovascular diseases.^[4,5]^ Particularly in older adults, the incidence of periodontitis is high, with approximately 80% of individuals over 60 years affected to varying degrees.^[6]^ Furthermore, periodontitis not only compromises oral health but is also linked to systemic health problems, including cardiovascular diseases, diabetes, and respiratory infections, all of which significantly reduce the patient’s quality of life.^[7]^ Therefore, early detection, effective treatment, and comprehensive management of periodontitis in the elderly are critical.^[8]^

Traditional treatments for periodontitis typically focus on localized interventions, such as scaling, subgingival debridement, and gingivectomy. While these treatments can alleviate localized symptoms, they often overlook the broader health needs of patients, particularly their long-term health management.^[9]^ Elderly patients, with their reduced immune function and multiple health conditions, often fail to achieve optimal outcomes from localized treatments alone.^[10]^ As a result, a more integrated and personalized care approach is needed to promote the overall recovery of elderly patients with periodontitis.

In recent years, the information, knowledge, attitude, practice (IKAP) nursing model has emerged as a promising health promotion strategy, gaining attention in clinical nursing practice.^[11]^ The IKAP model emphasizes the delivery of information, dissemination of knowledge, transformation of patient attitudes, and enhancement of practical skills to improve overall health literacy, promote positive health behaviors, and ultimately improve health outcomes.^[12]^ This approach not only enhances patients’ awareness of their condition but also fosters improvements in their health behaviors and lifestyle, thereby enhancing self-management and treatment adherence.^[13]^

Specifically, the IKAP model promotes health through 4 key interventions: First, by providing relevant information, patients gain a deeper understanding of periodontitis – its causes, risks, prevention, and treatment. Second, health education enables patients to acquire effective oral hygiene skills, encouraging the adoption of good oral care habits. Third, by shifting patients’ attitudes, the model enhances their self-care awareness and compliance with treatment. Finally, the model encourages patients to apply their health knowledge to everyday practices, boosting their motivation to engage in both treatment and preventive measures.^[14]^ Through these steps, the IKAP nursing model effectively improves patients’ health behaviors, reduces the incidence and complications of periodontitis, and slows disease progression.^[15]^

The IKAP nursing model holds significant potential for elderly patients with periodontitis, whose oral hygiene often deteriorates due to age-related physical changes, leading to oral health issues such as periodontitis.^[16]^ Moreover, elderly individuals frequently have multiple chronic conditions and weakened immune systems, making them more vulnerable to the exacerbating effects of periodontitis.^[17]^ Therefore, comprehensive care that combines localized treatment with overall health management and lifestyle interventions is essential. The IKAP model can enhance elderly patients’ oral hygiene habits by improving their understanding of the disease and encouraging healthier behaviors, ultimately helping to control the progression of periodontitis and improve their quality of life.^[2]^

The IKAP model aligns closely with core concepts of behavior change theory, particularly the Health Belief Model and the Transtheoretical Model. These theories emphasize that improving individuals’ knowledge and perceptions of health risks can promote attitude shifts and ultimately lead to sustained behavioral change. The IKAP model follows a similar logic – by first delivering relevant health information, then enhancing understanding (knowledge), shifting health beliefs (attitude), and promoting practical application (practice). This stepwise process reflects the psychological and behavioral mechanisms involved in adopting and maintaining healthy behaviors, making IKAP a theory-driven approach to health promotion.

While the IKAP model has been widely applied in the health promotion of chronic conditions such as hypertension, diabetes, and chronic respiratory diseases,^[18]^ there remains a lack of research on its application in elderly periodontitis patients, especially in terms of health promotion. Therefore, the objective of this study is to evaluate the effectiveness of the IKAP nursing model in elderly patients with periodontitis, particularly in terms of oral health improvement, disease control, and quality of life enhancement.^[19]^

This study will utilize a retrospective case-control design to assess the impact of the IKAP nursing model on elderly periodontitis patients by comparing it with traditional nursing methods. The findings will not only help validate the efficacy of the IKAP model but also provide new theoretical insights and practical experience for the care of elderly patients with periodontitis. Ultimately, this research aims to contribute to the development of a more scientific and systematic nursing model for managing elderly periodontitis patients, improving their oral health, and enhancing their overall quality of life, thereby supporting the health management needs of an aging society. It is hypothesized that the IKAP nursing model will result in greater improvements in oral health, disease control, and quality of life compared to traditional care models.

## 2. Methodology and analyses

### 2.1. Study design

This study was approved by the Ethics Committee of Shanghai East Hospital, School of Medicine (Ethical Approval Number: [SEH2022-041]). This study utilized a retrospective case-control study design with the aim of evaluating the effectiveness of the IKAP model of care in the health promotion of elderly patients with periodontitis. All data were treated confidentially and used solely for medical research purposes.

### 2.2. Study population

The study population consisted of elderly patients who were treated in our hospital between June 2022 and June 2024 with a diagnosis of periodontitis. A total of 131 patients were included in this study, with 63 patients in the conventional care group and 68 patients in the IKAP care group. Patients were retrospectively assigned to groups based on the type of nursing care they received during their clinical treatment period. This was a non-randomized design, and no formal matching was performed; however, baseline characteristics such as age, gender, disease stage, and comorbidities were statistically comparable between the 2 groups (*P* > .05), ensuring group comparability.

Inclusion criteria: age ≥ 60 years; periodontitis (including gingival bleeding, tartar buildup, etc) diagnosed by a dentist; patients were able to understand and cooperate with the study; participants were willing to cooperate with health education and follow-up.

Exclusion criteria: suffering from severe periodontal diseases (e.g. acute periodontal abscess); presence of history of oral and maxillofacial surgery; suffering from severe comorbidities (e.g. heart disease, diabetes mellitus, etc, which may affect the effectiveness of the nursing interventions); and severe cognitive disorders, which make it impossible for the participant to understand or cooperate with the nursing interventions.

### 2.3. Nursing care model

#### 2.3.1. Routine nursing care model

Basic health education: provide basic oral hygiene education, which includes brushing skills, flossing use, and so on. Education is usually conveyed through verbal explanations and simple promotional materials.

Routine oral care: routine oral care, such as gingival massage, oral cleaning, etc, was provided, but no personalized care interventions were provided based on the patient’s specific periodontal health status. Patients followed routine instructions for brushing, flossing, etc, but no specific action plan or regular supervision was involved.

Regular follow-ups and checkups: Regular follow-ups and dental checkups are performed to routinely clean and remove tartar, etc, based on the patient’s periodontal condition. The frequency of follow-up visits is usually more regular, but lacks personalized guidance and promotion of active patient participation.

Basic psychological support: Simple psychological support is provided to help patients understand the basics of periodontitis and the importance of treatment, but there are usually no specialized psychological interventions or emotional management measures. The content of psychological support was simplified, focusing on basic treatment coordination, and did not provide specialized coping strategies for possible anxiety or depression in older patients.

Patient self-care instructions: Basic self-care instructions were provided to patients, focusing on oral hygiene and dietary advice, but did not cover individualized self-management skills or long-term health maintenance strategies. It relies on patients to implement it on their own and lacks regular supervision and feedback.

Treatment adherence monitoring: patient adherence is monitored through regular follow-up visits and oral examinations, but regular reminders, support, or specific behavioral promotion measures are not provided. Patient adherence monitoring is lax and lacks specific interventions and behavioral guidance.

#### 2.3.2. IKAP model of care

Personalized nursing assessment: before the intervention, a detailed nursing assessment was conducted for each patient, which included oral hygiene status, severity of periodontitis, the patient’s level of knowledge of health management, and lifestyle. Nursing assessment was accomplished through questionnaires (including a self-made oral hygiene assessment form) and oral examinations. The assessment results were used to develop a personalized care plan to ensure that nursing interventions were matched to the specific needs of the patient.

Individualized health education: Based on the assessment results, an individualized health education plan was developed, which included: proper oral hygiene (e.g., brushing techniques, flossing, etc); knowledge of prevention and management of periodontitis; advice on healthy diet and lifestyle; and the importance of dental treatment and posttreatment precautions. Educational methods include face-to-face individualized instruction, group discussion, and distribution of pictorial materials.

Health behavior promotion: patients are encouraged to perform daily oral care according to their personal action plans, with goals including regular brushing, flossing, and regular dental checkups. Patients are regularly urged to implement the health management plan and provided with behavioral support through telephone follow-up and SMS reminders.

Psychological support and self-management training: Psychological counseling was organized to help patients cope with psychological stress caused by periodontitis, such as anxiety, depression and other emotional distress; self-management training was provided, including stress management and disease management skills, to enhance patients’ confidence and autonomy in health management.

During the implementation process, all caregivers undergo standardized training to ensure consistency and effectiveness of the intervention. The main components of the intervention will be carried out at 1, 3 and 6 months after the start of the intervention, and will be personalized according to the specific conditions of the patients.

### 2.4. Assessment indicators

The main assessment indicators of this study include:

1. Periodontal health improvement: clinical examination and periodontal probing were used to assess patients’ gingival health, including gingival index (GI), probing depth (PD), clinical attachment level (CAL), calculus and plaque index (CPI) and TM. The patients were compared before and after the assessment to see if their oral health had improved.
2. Behavioral stage change: the behavioral stage change scale was used to measure the change in oral health care behaviors of patients with periodontitis after treatment.
3. Oral health self-efficacy: the self-efficacy scale for self-care (SESS) was used to assess the self-efficacy of patients.
4. Quality of life: The oral health-related quality of life scale (OHIP-14) was used to assess the quality of life of the patients, especially the impact of oral health on daily life.

### 2.5. Data collection methods

Data collection will be carried out in the following ways:

1. Clinical examination: patients will be examined orally by a dentist to record the periodontal health status (including gingival bleeding, loose teeth, oral hygiene status, etc).
2. Questionnaire: patients completed the relevant scale at enrollment and during follow-up.
3. Follow-up records: patients’ self-care, compliance and health behaviors were recorded through telephone follow-up and outpatient follow-up.
4. Electronic health record: basic demographic information and relevant clinical data (e.g., past medical history, comorbidities, etc) were collected from the patients.

To improve the accuracy of the data, all follow-up visits will be conducted at predetermined time points, and a dedicated researcher will be responsible for recording patient compliance. Patient adherence will be assessed through a combination of dental follow-up records and self-reports. All data will be treated confidentially to ensure medical research use only.

### 2.6. Statistical analysis

Data analysis was performed using SPSS statistical software (version 26.0, Chicago). The specific steps were as follows: descriptive statistics: descriptive statistics of the basic characteristics of patients (such as age, gender, etc) were performed, using the mean ± standard deviation (Mean ± SD) or frequency (n); intergroup comparisons: for continuous variables, the independent samples *t*-test was used to compare the differences between 2 groups; for categorical variables, the chi-square test (χ² test) was used to analyze the differences between the 2 groups; for data that were not normally distributed data, nonparametric comparisons were made using the Mann–Whitney U test; significance level: the significance level was set at *P* < .05.

## 3. Results

### 3.1. Baseline characteristics

A total of 131 elderly patients with periodontal disease were enrolled in this study, and were divided into the IKAP care group (68) and the routine care group (63) according to the different modes of care during the consultation process. There were no significant differences between the 2 groups in terms of age, gender, previous occupation, monthly household income, type of medical payment, family history of oral disease, periodontal disease stage, smoking, and alcohol consumption (*P*-value > .05), ensuring comparability of baseline characteristics between the 2 groups. See Table 1 for details.

**Table 1:** Comparison of general data of patients in the 2 groups.

	IKAP care (n = 68)	Conventional care (n = 63)	*t*/χ2	*P*-value
Age	67.32 ± 6.33	66.23 ± 5.41	1.06	.29
Gender				
Male	45	44	0.39	.49
Female	23	19		
Previous occupation				
Farmer	28	25	1.08	.11
Professionals and technicians	15	17		
Self-employed	10	8		
Civil servant	12	9		
Other	3	4		
Monthly household income				
Below 5000	29	28	1.39	.15
5000–10,000	27	20		
Above 10,000	12	15		
Type of medical payment				
Medicare	44	39	0.64	.27
Self-pay	14	12		
Other	10	12		
Family history of oral disease				
Yes	38	34	0.24	.57
No	30	29		
Periodontal disease stage				
Mild	17	21	1.03	.18
Moderate	29	26		
Severe	20	16		
Smoking				
Yes	39	37	0.22	.56
No	29	26		
Drinking				
Yes	41	42	0.76	.35
No	27	21		

IKAP = information–knowledge–attitude–practice.

### 3.2. Improvement in periodontal health

#### 3.2.1. Gingival index (score)

At the 1-month and 3-month follow-up visits, the differences in GI between the IKAP care group and the usual care group were not significant. The difference in GI between the IKAP care group and the usual care group did not reach statistical significance at 1 month (*t* = 0.31, *P* = .57), nor was the difference statistically significant at 3 months (*t* = 2.17, *P* = .14). However, at 6 months, the patients in the IKAP care group showed a difference from the patients in the usual care group, which reached statistical significance (*t* = 4.90, *P* = .027), suggesting that IKAP care model significantly reduced gingival bleeding over a 6-month period.

#### 3.2.2. Probing depth (mm)

In the comparison of PD at different time points, the difference between the IKAP care group and the usual care group did not reach statistical significance at 1 and 3 months (1 month: *t* = 0.55, *P* = .57; 3 months: *t* = 0.83, *P* = .40). However, at 6 months, the PD was significantly lower in the IKAP care group (4.21 ± 2.01 mm) than in the usual care group (5.01 ± 2.43 mm), and the difference was statistically significant (*t* = 2.05, *P* = .04).

#### 3.2.3. Clinical attachment level

In the comparison of CALs, the difference between the IKAP care group and the usual care group did not reach statistical significance at both 1 and 3 months (1 month: *t* = 0.85, *P* = .39; 3 months: *t* = 1.35, *P* = .17). However, at 6 months, the level of clinical attachment was significantly better in the IKAP care group (3.56 ± 2.15 mm) than in the usual care group (4.52 ± 2.66 mm), and the difference was statistically significant (*t* = 2.27, *P* = .02).

#### 3.2.4. Calculus and plaque index (score)

At the 1-month and 3-month follow-up visits, the differences in CPI between the IKAP care group and the usual care group were not significant. The difference in CPI between the IKAP care group and the usual care group did not reach statistical significance at 1 month (*t* = 0.85, *P* = .35), nor was the difference statistically significant at 3 months (*t* = 0.14, *P* = .89). However, at 6 months, patients in the IKAP care group showed a difference from patients in the usual care group, which reached statistical significance (*t* = 5.263, *P* = .022).

#### 3.2.5. Tooth mobility (score)

Comparison of TM showed that the IKAP care group did not exhibit significant differences with the conventional care group at 1 month (*t* = 0.33, *P* = .56), 3 months (*t* = 2.05, *P* = .17), and 6 months (*t* = 0.49, *P* = .484) of follow-up. The specific data analyzed are shown in Table 2.

**Table 2:** Improvement of periodontal health in 2 groups of patients.

	1 mo				3 mo				6 mo			
	IKAP care	Conventional care	*t*/χ2	*P*-value	IKAP care	Conventional care	*t*/χ2	*P*-value	IKAP care	Conventional care	*t*/χ2	*P*-value
GI (score)												
1	16	13	0.31	.57	28	18	2.17	.14	42	24	4.90	.027
2	35	32			30	29			23	28		
3	17	18			10	16			3	11		
PD (mm)	5.51 ± 2.39	5.33 ± 2.09	0.55	.57	4.89 ± 2.55	5.23 ± 2.09	0.83	.40	4.21 ± 2.01	5.01 ± 2.43	2.05	.04
CAL (mm)	4.52 ± 2.23	4.85 ± 2.19	0.85	.39	4.11 ± 2.64	4.71 ± 2.41	1.35	.17	3.56 ± 2.15	4.52 ± 2.66	2.27	.02
CPI (score)												
1	26	22	0.85	.35	35	28	0.14	.89	42	32	5.26	.022
2	30	26			24	22			21	20		
3	12	15			9	13			5	11		
TM (score)												
1	16	16	0.33	.56	17	18	2.05	.17	21	15	0.49	.484
2	34	31			36	31			36	37		
3	18	16			15	14			11	11		

CAL = clinical attachment level, CPI = calculus and plaque index, GI = gingival index, IKAP = information–knowledge–attitude–practice, PD = probing depth, TM = tooth mobility.

### 3.3. Changes in behavioral stages

In terms of changes in behavioral stages, the differences between the IKAP care group and the conventional care group were statistically significant at 1 month, 3 months and 6 months. In particular, at 6 months, more patients in the IKAP care group were in the “action stage” and “maintenance stage,” while patients in the usual care group were in the “intention stage” or “preparation stage.” At 6 months, the number of patients in the “action stage” was 17 in the IKAP care group compared with 4 in the usual care group, a significant difference (χ² = 13.213, *P* = .01), suggesting that IKAP care helps to promote the adoption and maintenance of health behaviors. See Table 3 for details.

**Table 3:** Changes in behavioral stages of patients in 2 groups.

	1 mo				3 mo				6 mo			
	IKAP care	Conventional care	*t*/χ2	*P*-value	IKAP care	Conventional care	*t*/χ2	*P*-value	IKAP care	Conventional care	*t*/χ2	*P*-value
Behavioral stages												
Pre-intent stage	34	30	1.8	.772	16	28	8.16	.086	11	20	13.21	.01
Intent stage	11	15			18	16			15	18		
Preparation stage	17	15			23	15			16	18		
Action stage	4	2			8	3			17	4		
Maintenance stage	2	1			3	1			8	3		

IKAP = information–knowledge–attitude–practice.

### 3.4. Oral health self-efficacy

#### 3.4.1. Oral visits

At all time points, the IKAP care group scored significantly higher on oral visits than the usual care group. At 1, 3, and 6 months, the IKAP care group scored 21.54, 21.33, and 20.89, respectively, compared to 19.69, 19.42, and 18.97, respectively, for the usual care group, with the IKAP care group consistently scoring significantly higher than the usual care group (all *t*-values > 5.0, *P*-values < .01).

#### 3.4.2. Correct tooth brushing

The IKAP care group also scored significantly better than the usual care group in terms of proper brushing of teeth. 21.67, 21.32, and 20.89 at 1, 3, and 6 months, respectively, compared to 19.78, 19.56, and 19.02, respectively, for the usual care group, with the IKAP care group scoring significantly higher than the usual care group (t-value > 5.0 for all, *P*-value < .01).

#### 3.4.3. Balanced diet

In terms of balanced diet, the IKAP care group also consistently scored higher than the usual care group. The IKAP care group scored 22.46, 22.29, and 21.74 at 1, 3, and 6 months, respectively, compared to 20.23, 20.01, and 19.65 in the usual care group, and the scores in the IKAP care group were significantly higher than those in the usual care group (all *t*-values > 5.0, all *P*-values < .01). See Table 4 for details.

**Table 4:** Oral health care self-efficacy of patients in the 2 groups.

	oral visits			Correct tooth brushing			Balanced diet		
	1 mo	3 mo	6 mo	1 mo	3 mo	6 mo	1 mo	3 mo	6 mo
IKAP care (n = 68)	21.54 ± 2.06	21.33 ± 2.33	20.89 ± 2.01	21.67 ± 2.24	21.32 ± 1.96	20.89 ± 1.88	22.46 ± 1.79	22.29 ± 1.88	21.74 ± 2.04
Conventional care (n = 63)	19.69 ± 2.03	19.42 ± 2.06	18.97 ± 2.14	19.78 ± 2.08	19.56 ± 2.06	19.02 ± 2.15	20.23 ± 2.04	20.01 ± 2.42	19.65 ± 2.34
*t*	5.17	6.23	5.01	5.89	5.78	5.11	5.17	6.12	6.27
*P*-value	<.01	<.01	<.01	<.01	<.01	<.01	<.01	<.01	<.01

IKAP = information–knowledge–attitude–practice.

### 3.5. OHIP-14 total score

The IKAP care group consistently scored significantly lower on the OHIP-14 total score than the conventional care group, indicating that IKAP care had a greater impact on improving patients’ oral health. The scores of the IKAP care group at 1 month, 3 months, and 6 months were 14.69, 15.58, and 16.89, respectively, compared to 11.32, 13.33, and 13.26, respectively, for the conventional care group, and the IKAP scores were significantly higher in the usual care group (all t-values > 3, p-values < 0.05). See Table 5 for details.

**Table 5:** Oral health care self-efficacy of patients in the 2 groups.

		OHIP-14 total score	*t*	*P*-value
1 mo				
IKAP care (n = 68)	–	14.69 ± 5.33	3.12	.03
Conventional care (n = 63)		11.52 ± 5.49		
3 mo				
IKAP care (n = 68)	–	15.58 ± 4.32	3.24	.02
Conventional care (n = 63)		12.33 ± 6.95		
6 mo				
IKAP care (n = 68)	–	16.89 ± 7.04	3.78	.02
Conventional care (n = 63)		13.26 ± 4.55		

IKAP = information–knowledge–attitude–practice, OHIP-14 = oral health impact profile-14.

## 4. Discussion

As the global elderly population continues to grow, health issues, particularly oral health problems, are becoming more prominent. Periodontitis, a common chronic oral disease, has become a significant concern among older adults. This condition not only directly impacts their oral health, leading to tooth loss and difficulty in chewing, but can also affect their overall health and quality of life.^[20,21]^ With advancing age, factors such as weakened immune function, frequent medication use, and poor lifestyle habits increase the vulnerability of older adults to periodontitis.^[22]^

Nursing interventions play a crucial role in managing periodontitis in the elderly. The IKAP (Individualized Knowledge and Action Plan) nursing model has emerged as a promising approach for the care of patients with chronic diseases, delivering personalized care with remarkable results.^[23]^ This model focuses on tailored health education, behavioral interventions, and continuous health management, aimed at enhancing patient compliance and improving life quality.^[24]^

This study demonstrates that applying the IKAP model in elderly patients with periodontitis significantly improves oral health, quality of life, and disease management adherence. Compared to traditional care models, the IKAP model offers more personalized interventions that help patients better understand and manage their oral health. Through a combination of health education, behavioral changes, and ongoing support, the IKAP model effectively promotes active patient participation, leading to improved periodontal health and quality of life.

A core feature of the IKAP model is its emphasis on engaging patients through personalized education and action plans. This helps patients gain a better understanding of their condition, manage it more effectively, and adopt healthier behaviors.^[25]^ The study revealed significant improvements in the periodontal index and gingival bleeding among those using the IKAP model, consistent with prior research.^[26]^ Unlike traditional care models, which often focus solely on disease treatment, the IKAP model places a greater emphasis on behavioral changes and lifestyle improvements. Traditional approaches typically limit themselves to treatment without providing in-depth, personalized interventions.^[27]^ In contrast, the IKAP model helps patients establish healthier behaviors through regular education and psychological support, thereby improving treatment adherence and outcomes.^[28]^

Health education is a central component of the IKAP model. In this study, health education covered not only basic oral health knowledge but also lifestyle habits, dietary control, and psychological adjustment. Through continuous education, patients’ understanding of periodontitis significantly improved, leading to more active participation in managing their condition.^[29]^ Elderly patients often have limited knowledge and require more guidance, and the IKAP model provides long-term, tailored support to help them develop self-care skills.^[30]^ Moreover, behavioral interventions are essential to the IKAP model. In this study, patients showed improvements in lifestyle habits, such as reducing smoking and enhancing oral hygiene, due to regular behavioral interventions. This highlights the comprehensive, individualized nature of the IKAP model.^[31]^

The IKAP model also emphasizes regular follow-up and continuous support to maintain health status during the treatment process. In this study, follow-ups were primarily conducted through phone calls and SMS to remind patients about appointments and the importance of maintaining oral hygiene.^[32]^ This improved treatment compliance and fostered better communication between patients and caregivers. Research has shown that patients receiving IKAP interventions exhibit higher adherence to treatment and better disease control during follow-ups.^[33]^

Although this study highlights the positive outcomes of the IKAP model in elderly periodontitis patients, there are several limitations. First, the study design was a retrospective case-control with a small sample size and a single sample source, which may have introduced selection bias. Second, long-term follow-up was not conducted to assess the lasting effects of the IKAP model. Future studies should address these limitations by conducting larger prospective randomized controlled trials to validate the generalizability and long-term effectiveness of the IKAP model.

Additionally, exploring the impact of the IKAP model on the mental health of elderly patients with periodontitis could provide further insights. Many elderly patients experience psychological issues such as loneliness and anxiety, which could influence their acceptance and adherence to nursing interventions. Future research could integrate psychological support into the IKAP model to evaluate its effect on improving both physical and mental health.

The successful application of the IKAP nursing model offers a new perspective for clinical practice, particularly in elderly patient care. Clinicians can tailor care plans to individual patient needs, provide ongoing health education and behavioral interventions, and help patients improve both their oral health and overall quality of life. Furthermore, the IKAP model does not require high healthcare costs, making it an effective approach for primary healthcare settings, especially in resource-limited areas.

This study has several limitations. First, the retrospective case-control design may introduce selection bias, and the non-randomized grouping limits the ability to draw strong causal inferences. Although baseline characteristics between groups were statistically comparable, potential unmeasured confounders may still exist. Second, the study was conducted in a single hospital, and the sample size was relatively small, which may limit the generalizability of the findings. Third, the intervention period and follow-up duration were limited to 6 months. Although this period allowed for the observation of mid-term effects, it was insufficient to assess long-term outcomes such as the maintenance of behavioral changes and recurrence of periodontal disease. Therefore, this study should be considered a preliminary observation of the short-term effectiveness of the IKAP model. Future prospective, multicenter studies with larger sample sizes and extended follow-up periods of 12 months or more are recommended to validate these findings and explore the long-term impact of the intervention.

The findings of this study suggest that the IKAP nursing model significantly improves the oral health and quality of life of elderly periodontitis patients. Through personalized health education, behavioral interventions, and continuous follow-up, the IKAP model enhances treatment adherence and promotes better oral health. Despite the positive results, future studies are needed to address the current limitations. The widespread application of the IKAP model in elderly periodontitis care could greatly contribute to improving the overall health of elderly patients.

## Author contributions

**Conceptualization:** Yubo Xu, Yeqian Zhu.

**Data curation:** Yubo Xu, Yeqian Zhu.

**Formal analysis:** Yubo Xu, Yeqian Zhu.

**Investigation:** Yubo Xu, Jing Qian.

**Methodology:** Yubo Xu, Jing Qian, Guojun Miao.

**Supervision:** Yubo Xu, Jing Qian, Guojun Miao.

**Validation:** Yubo Xu, Wen Ma, Yeqian Zhu.

**Visualization:** Yubo Xu, Wen Ma, Yeqian Zhu.

**Writing** – **original draft:** Yubo Xu, Yeqian Zhu.

**Writing** – **review & editing:** Yubo Xu, Yeqian Zhu.
